# Standardizing the Assessment of Macular Pigment Using a Dual-Wavelength Autofluorescence Technique

**DOI:** 10.1167/tvst.8.6.41

**Published:** 2019-12-18

**Authors:** Marina Green-Gomez, Paul S. Bernstein, Christine A. Curcio, Rachel Moran, Warren Roche, John M. Nolan

**Affiliations:** 1 Nutrition Research Centre Ireland, School of Health Science, Carriganore House, Waterford Institute of Technology, West Campus, Waterford, Ireland; 2 Moran Eye Center, University of Utah, Salt Lake City, UT, USA; 3 Department of Ophthalmology and Visual Sciences, University of Alabama at Birmingham, Birmingham, AL, USA

**Keywords:** macular pigment, lutein, zeaxanthin, dual-wavelength autofluorescence

## Abstract

**Purpose:**

It is essential to have an appropriate measure to assess macular pigment (MP) that can provide an accurate, valid, and reliable representation of the MP within the macula. The aim of this study was to describe and introduce MP optical volume (MPOV) as an optimal value for reporting MP.

**Methods:**

Three hundred ninety-three subjects were analyzed using the Heidelberg Spectralis with the investigational MP optical density (MPOD) module to measure MPOV and MPOD at four foveal eccentricities (0.23°, 0.51°, 0.98°, 1.76° [7° as reference point]). Lutein (L) and zeaxanthin (Z) dietary intake and serum concentrations were evaluated.

**Results:**

MPOV mean was 5094 (95%CI, 4877–5310); range: 527 to 10,652. MPOV was inversely correlated with body mass index and positively correlated with education (*r* = −0.156, *P* = 0.002 and *r* = 0.124, *P* = 0.014, respectively). Serum concentrations of L and Z were positively correlated with MPOV (*r* = 0.422, *P* < 0.001 and *r* = 0.285, *P* < 0.001, respectively). MPOV was positively correlated to MPOD at all measured eccentricities, with the strongest agreement at 1.76° (*r* = 0.906, *P* < 0.001). Serum concentrations of L and Z, BMI, education, and age (*P* < 0.001) were found to be significant predictors of MPOV.

**Conclusions:**

The Spectralis MPOV measurement provided a comprehensive and detailed evaluation of the MP profile. The Spectralis MPOV should be considered a preferred metric for the assessment of MP.

**Translational Relevance:**

Applying a standardized method for the assessment and report of MP will allow to fully derive meaning from observational studies and to successfully implement this MP measurement technique in research and clinical settings.

## Introduction

Lutein (L), zeaxanthin (Z), and meso-zeaxanthin (MZ)^1^,^2^ are natural lipid-soluble pigments belonging to the *xanthophyll* class of the carotenoid family,^3^ which are entirely of dietary origin.^4^ Research into the role of these carotenoids has intensified over the last two decades, due to their role in human health, with the eye having been the focus of major research.^5^

L, Z, and MZ are concentrated in the macula,^6^ where they are collectively referred to as macular pigment (MP). The macula is the central region of the human retina approximately 5.5 mm in diameter,^6^ with three concentric subregions from the center outward, the fovea, surrounded by annuli of parafovea, and the perifovea. This region has multiple tiers of retinal ganglion cells, which are reduced to one row at the outer boundary, and are absent from the foveal depression.^7^ The macula, which mediates high-visual acuity^8^ and color vision,^9^ exhibits a regional susceptibility^10^ to diseases that impact negatively on visual performance and vision-related quality of life.^11^–^13^ Of note, the characteristics of MP, which include antioxidant,^14^ anti-inflammatory,^15^ and optical properties,^16^ are ideally suited to protect this tissue against oxidative stress and inflammation. In addition, some intervention studies with L and Z supplementation have demonstrated a reduced risk of disease progression in patients with nonadvanced age-related macular degeneration (AMD),^17^ and other studies with the addition of MZ have shown improvement in visual function.^18^ Moreover, recent studies have shown MP to be positively correlated with brain carotenoid levels^19^,^20^ and cognitive function.^21^ Improvements in the latter have been demonstrated following supplementation with the three macular carotenoids,^22^ with potential implications for neurocognitive health and neurocognitive disorders (NCD), such as Alzheimer's disease (AD).^23^

Numerous published studies that report on MP have used a variety of measurement techniques, all with different interpretations and with their own assumptions and limitations, and consequently, it has been difficult to create normative databases. To understand fully the impact of MP on macular health and function, the establishment of a reference database for MP is essential so that treatment effects can be closely monitored. For example, this would have been useful in the AREDS2 study,^17^ which has become the standard of care without the benefit of real-time monitoring of MP in retinal tissues during the trial.

Existing techniques that measure MP can be summarised as objective or subjective. Subjective techniques are typically psychophysical and include matching motion photometry and heterochromatic flicker photometry (HFP),^24^ with the latter being the most validated and widely used technique to date. Objective methods are imaging-based and include resonance Raman spectroscopy, reflectometry, and fundus autofluorescence (AF).^25^

For MP measurements to be used effectively and standardized in practice, it is essential to have an appropriate measure for reporting MP. Ideally, this measurement should be easy to obtain, accurate, reliable, and representative of the total amount and distribution of MP.

Hence, essential questions need to be answered for all available and employed techniques, including:

1. What techniques are available to measure MP?
2. What are the assumptions/limitations of these measurement techniques?
3. What are these techniques actually measuring?

Likewise, when interpreting MP measurement and reporting and selecting the appropriate technique to do so, crucial questions are almost compulsory to be understood:

1. What is MP optical density (MPOD)?
2. Is it sufficient to report MP at a central eccentricity only?
3. Is there value in reporting the profile of the pigment within a given eccentricity?
4. What is the current optimal method to measure MP?

In this paper we attempted to answer these questions for the Spectralis MPOD module. The aim of the current study was to introduce the term MP optical volume (MPOV), and to show the suitability of this metric for standardizing the assessment of MP detected by two-wavelength AF images.

## Methods

### Study Design and Sample

This was a cross-sectional study using data collected from a series of studies recently conducted at the Nutrition Research Centre Ireland. A total of 393 subjects, including 224 women and 169 men, with MP measurements obtained using the Spectralis MPOD module (vid infra) were included. The data presented in this report were collected between 2013 and 2017, and included the following variables: demographic characteristics, medical information, serum concentrations of L and Z, and MP measurements. All subjects satisfied individual study's regulations, complied fully with the tenets of the Declaration of Helsinki, and were granted ethical approval by local ethics committees at the Waterford Institute of Technology and South East Region. Participants aged 18 years and above with no critical medical condition were included. Previous history of oral carotenoid supplementation was considered an exclusion criterion. A total of 37% (*n* = 147) of the participants had the diagnosis of nonadvanced AMD, MCI, or AD; participants were included according to the following criteria: nonadvanced AMD is classified as 2 to 8 on the AREDS severity scale of color fundus photography (CFP),^26^ confirmed by the Moorfields Eye Hospital Reading Centre, London, UK; best-corrected visual acuity (BCVA) of 6 of 12 (20/40) or better in the study eye; and a refractive error of less than 5-dioptres spherical equivalent in the study eye. Patients with retinal pathology other than AMD, as determined on both CFP and optical coherence tomography (OCT), such as diabetes mellitus (by self-report), were excluded.

MCI is defined as memory complaints reported by family member or self-reported that fulfilled criteria for MCI in the Repeatable Battery for the Assessment of Neuropsychological Status (RBANS)^27^,^28^ and the Montreal Cognitive Assessment (MoCA)^24^; with functional independence in activities of daily living (as per the Bristol Activities of Daily Living Scale [BADLS]^29^,^30^). Participants were excluded if diagnosed with active or uncontrolled depression, or stroke.

Mild-to-moderate AD is defined as an average Mini-Mental State Examination (MMSE)^31^ score of 14 to 24 with documented difficulties carrying out everyday tasks assessed by caregivers, along with alteration in behavior, errors in the clock drawing test, and semantic fluency score. Participants were excluded if they were diagnosed with stroke.

Participants with no diagnosis of the aforementioned diseases were adjudged “healthy.”

### Study Evaluations

#### Demographic, Lifestyle, Medical, Ocular, and Dietary Assessment

Standardized case report forms were used to record demographics, lifestyle, and medical and family history. Consumption of carotenoid-rich foods (specifically eggs, broccoli, corn, dark leafy vegetables) was entered into a dietary L/Z screener offered by Tufts University, to provide a carotenoid-based diet score. The reference values of L and Z used in the screener were those reported by Perry et al.^32^ The resulting values were weighted for frequency of food intake and for L and Z bioavailability within these foods. A score ranging from 0 to 75 was allocated, and classified into three categories as follows: category 1, low intake (≤2 mg/d): score 0 to 15; category 2, medium intake (3–13 mg/d): score 16 to 30; and category 3, high intake (>13 mg/d): score 31 to 75. Cigarette smoking was recorded by smoking status as never, past, or current, and was evaluated by smoking risk with pack-year defined as the equivalent of smoking one pack of cigarettes (20 cigarettes) per day for 1 year. Education was recorded as primary, secondary, or third level of education. Physical examination included height and weight measurements, used to calculate body mass index (BMI in kg/m^2^).

#### Dual-Wavelength Autofluorescence-Based Macular Pigment Measurement

MPOV was the primary outcome variable in this study. It was measured by dual-wavelength AF using the Spectralis investigational macular pigment optical density (MPOD) module (Heidelberg Engineering GmbH, Heidelberg, Germany). Pupils were dilated prior to MP measurement, and patient details were entered into the Heidelberg Eye Explorer (HEYEX version 1.7.1.0) software. Alignment, focus and camera sensitivity were first optimized in near-infrared reflectance mode. Subsequently, simultaneous blue and green AF (BAF+GAF) movie images were acquired, while the HEYEX software ensured proper alignment and averaging of these images in order to generate a MP density map (details below). The MP density map, along with the subtracted MP AF attenuation image output and description of the analyzed MPOD point locations have been described elsewhere.^33^ In short, in order to generate a MP density map, the reference eccentricity has to be defined by the user, where MPOD is defined as zero. In this study, the reference point was set at 7° retinal eccentricity from point of fixation, for consistency and to allow for comparison with previous studies using subjective techniques to measure MP (Fig. 1). Equally, the averaged MPOD was recorded at four radii, 0.23° (central), 0.51°, 0.98°, and 1.76° eccentricities (concentric circles from the center). Likewise, these eccentricities were chosen corresponding to the eccentricities used by subjective techniques.^34^–^36^

**Figure 1:**
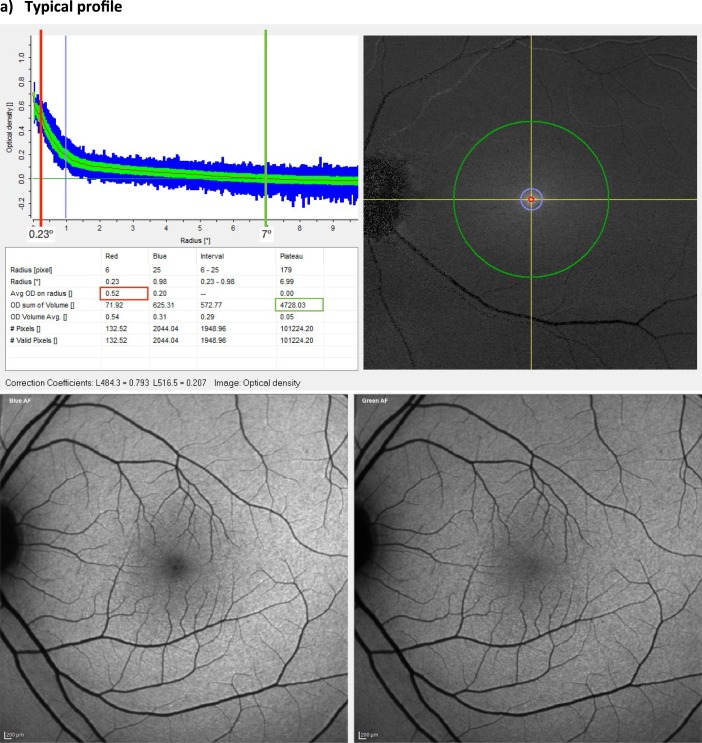
Macular pigment measurement. Spectralis MPOD module output. Comparison between two healthy subjects with equal MPOD at 0.23° of eccentricity (red line), showing subject (a) with low MPOV (green circumference) and subject (b) with high MPOV. Circumferences are the average MPOD of a circle of pixels centred on the fovea at each eccentricity (i.e., red circle at 0.23°, green circle at 7°) and green band is the standard deviation and the blue band is the range.

**Figure 1:**
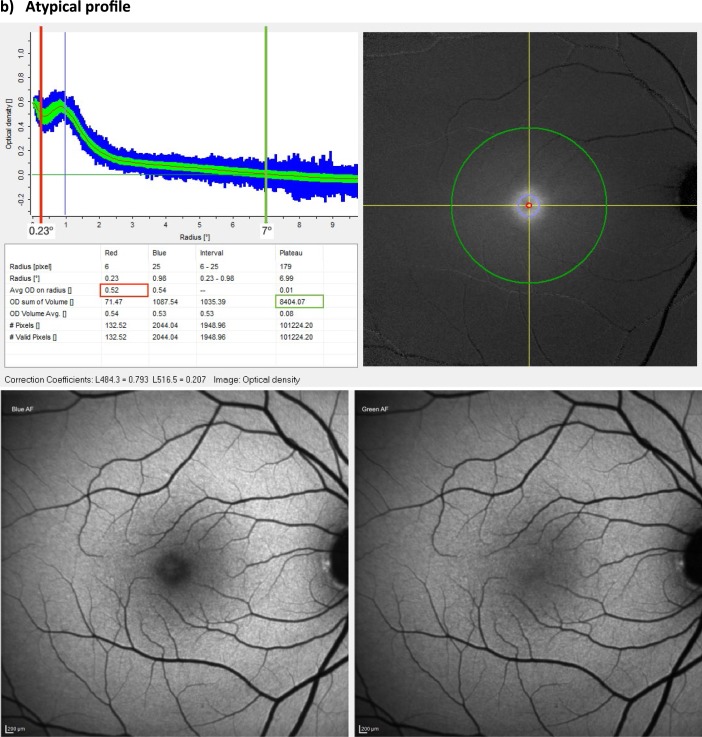
Continued.

#### MPOV Measurement

Technical specifications of MP dual-wavelength AF image acquisition and the computation of MPOD have been described in detail by Delori.^37^ In short, the Spectralis MPOD module uses confocal scanning laser ophthalmoscopy with blue (486 nm) and green (518 nm) laser diodes for AF excitation. Because MP is yellow, it absorbs light of its opposite primary color (blue) four times stronger than green light. Comparison of autofluorescence emission intensities generated by the retinal pigment epithelium (RPE) upon excitation by the two excitation wavelengths allows for MPOD measurements at any retinal location, via application of the Beer-Lambert Law. Optical density values refer to the inverse logarithm of the amount of transmitted light relative to the total amount of incident light as it passes through some material. MPOD is calculated as the log-ratio of green- relative to blue-excited AF intensities by the RPE. In the presence of any level of MP, the intensity of the blue light–excited AF should be lower than that of green light–excited AF at the same location. The Beer-Lambert law defines the log-ratio of both fluorescence emission light intensities to be MPOD:

\newcommand{\bialpha}{\boldsymbol{\alpha}}\newcommand{\bibeta}{\boldsymbol{\beta}}\newcommand{\bigamma}{\boldsymbol{\gamma}}\newcommand{\bidelta}{\boldsymbol{\delta}}\newcommand{\bivarepsilon}{\boldsymbol{\varepsilon}}\newcommand{\bizeta}{\boldsymbol{\zeta}}\newcommand{\bieta}{\boldsymbol{\eta}}\newcommand{\bitheta}{\boldsymbol{\theta}}\newcommand{\biiota}{\boldsymbol{\iota}}\newcommand{\bikappa}{\boldsymbol{\kappa}}\newcommand{\bilambda}{\boldsymbol{\lambda}}\newcommand{\bimu}{\boldsymbol{\mu}}\newcommand{\binu}{\boldsymbol{\nu}}\newcommand{\bixi}{\boldsymbol{\xi}}\newcommand{\biomicron}{\boldsymbol{\micron}}\newcommand{\bipi}{\boldsymbol{\pi}}\newcommand{\birho}{\boldsymbol{\rho}}\newcommand{\bisigma}{\boldsymbol{\sigma}}\newcommand{\bitau}{\boldsymbol{\tau}}\newcommand{\biupsilon}{\boldsymbol{\upsilon}}\newcommand{\biphi}{\boldsymbol{\phi}}\newcommand{\bichi}{\boldsymbol{\chi}}\newcommand{\bipsi}{\boldsymbol{\psi}}\newcommand{\biomega}{\boldsymbol{\omega}}\begin{equation}\tag{1}{\rm{MPOD}} = {\rm{Lo}}{{\rm{g}}_{10}}\left( {{{{I_{{\rm{Green}}}}(z)} \over {{I_{{\rm{Blue}}}}(z)}}} \right) \end{equation}

where *I*_Green_(*z*) = intensity of green-excited AF at z, *I*_Blue_(*z*) = intensity of blue-excited AF at *z,* and z = thickness of retinal boundaries within which MP is contained.

Based on these fundamentals, MPOV can be derived, whereby volume refers to the numeric integration of all MPOD values within a given area. MPOV thus represents the sum of all MPOD values for all pixels with valid results within the area delimited by the circumference of a chosen eccentricity. Given that MPOD is dimensionless, MPOV is hence unitless as well. Pixels without valid results are automatically identified by the HEYEX software and excluded from the analysis. These invalid pixel results are mainly caused by eye saccades, preventing appropriate alignment and hence processing of data between the blue and green AF images (Fig. 1).

#### Blood Sample Analysis: Serum Carotenoid (L, Z, and MZ) Concentrations

Nonfasting blood samples were collected by standard venipuncture techniques in 9-mL blood collection tubes (BD Vacutainer SST Serum Separation Tubes; Becton, Dickinson and Company, Plymouth, UK) containing a “Z Serum Sep Clot Activator.” Collection tubes underwent thorough mixing of the clot activator. The blood samples were left for 30 minutes at room temperature to clot and then centrifuged at 725 *g* for 10 minutes in a centrifuge to separate the serum from the whole blood. Following centrifugation, serum was transferred to light-resistant microtubes and stored at circa –80°C until the time of batch analysis. Serum carotenoid analysis was performed by high-performance liquid chromatography (HPLC), using a method that has been previously described.^38^

### Statistical Analysis

The statistical package IBM SPSS version 25 (Armonk, NY) was used, and a 5% significance level was applied. The dependant variable MPOV did not demonstrate a typical normal distribution. Descriptive statistics included means, standard deviation (SD), and median, interquartile range (IQR), minimum, and maximum values for quantitative variables. Between-group differences were analyzed using Kruskal-Wallis H Test. Spearman's rank coefficient was used to investigate potential relationships between MPOV and age, education, BMI, serum concentrations, and MPOD at all eccentricities. Data were then analyzed using linear regression analysis.

## Results

The mean age (±SD) of the 393 participants was 56 (±15) years (Table 1). Sixty-three percent of the participants were considered healthy (*n* = 246) and the other 37% (*n* = 147) had a medical diagnosis (AMD or a neurocognitive disorder, MCI or AD).

**Table 1:** MPOV, Clinical, and Demographic Characteristics

Variable	Subjects (*n* = 393)
MPOV	
Mean	5094
(95%CI)	(4877–5310)
Median	4893
IQR	3588–6524
Age (y), mean (SD)	55.8 (15.5)
Range	21–88
Females, *N* (%)	224 (57.0)
Level of education, *N* (%)^a^	
Primary	36 (10.2)
Secondary	146 (41.2)
Higher	172 (48.6)
L/Z diet score^b^	
Median (IQR)	21 (10–28)
Range	0–62
Smoking status, *N* (%)	
Nonsmoker	208 (52.9)
Former	143 (36.4)
Current	42 (10.7)
Health risk: pack-year^c^	
Mean (SD)	6.2 (12.8)
Diagnosis, *N* (%)	
No known disease	246 (62.6)
AMD	115 (29.3)
MCI	18 (4.6)
AD	14 (3.5)
BMI, Mean (SD)	27.3 (4.8)
Serum L, μmol/L	
Mean (SD)	0.244 (0.156)
95%CI	0.228–0.260
Median	0.201
Minimum–maximum	0.046–1.20
IQR	0.142–0.291
Serum Z, μmol/L	
Mean (SD)	0.078 (0.052)
95%CI	0.073–0.084
Median	0.064
Minimum–maximum	0.012–0.393
IQR	0.047–0.092

a Data available for 257 participants.

b Lutein and Zeaxanthin dietary intake (data available for 296 participants): low intake (≤2 mg/day): score 0–15; medium intake (3–13 mg/day): score 16–30; and high intake (>13 mg/day): score 31–75.

c A pack-year is defined as the equivalent of smoking one pack of cigarettes (20 cigarettes) per day for 1 year calculated for all participants.

### MPOV Characteristics

MPOV mean was 5094 (95%CI, 4877–5310) with minimum and maximum values of 527 and 10,652, respectively, demonstrating the large variability between individuals (Table 1). Healthy subjects had a mean MPOV (95%CI) of 5094 (4796–5326); subjects diagnosed with AMD, MCI, and AD had a mean MPOV (95%CI) of 5190 (4767–5612), 5687 (4450–6924), and 4131 (2912–5350), respectively. After controlling for confounding variables, comparison of MPOV between medical diagnoses (i.e., heathy, AMD, MCI, and AD) demonstrated no significant difference (*P* = 0.096). Hence, the sample was considered as one group for further analyses when establishing predictors and correlations of MPOV.

**Figure 2:**
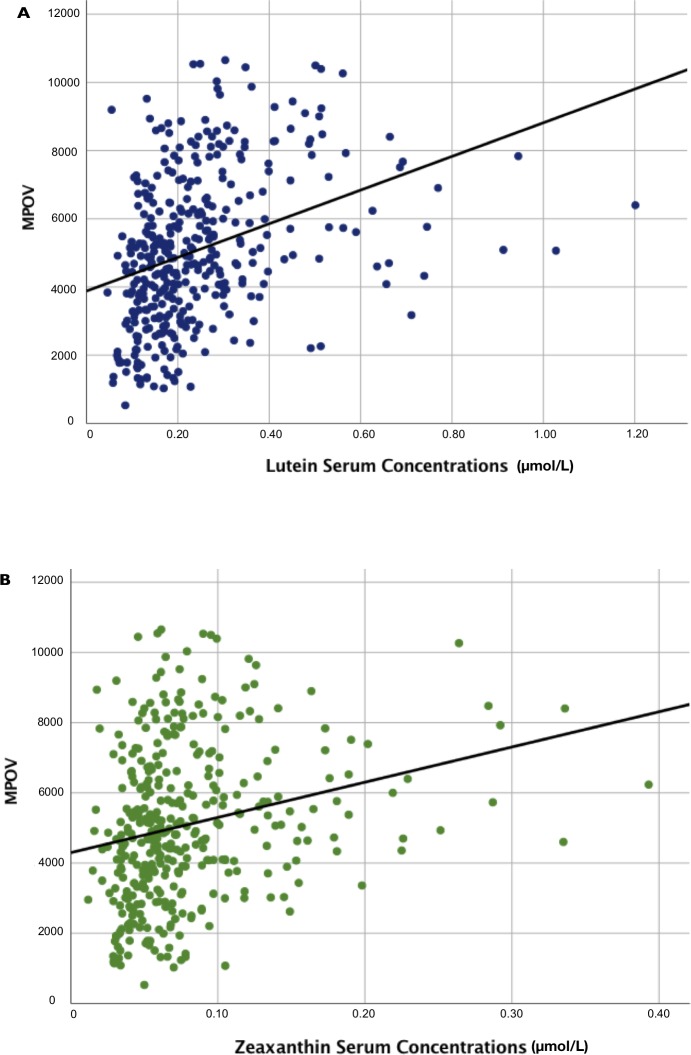
The relationship between MPOV and serum concentrations of lutein and zeaxanthin. Linear regression analyses of MPOV and (a) serum lutein (r = 0.422, P < 0.001), and (b) zeaxanthin (r = 0.285, P < 0.001).

MPOV was inversely correlated with BMI and positively correlated with education level (*r* = −0.156, *P* = 0.002 and *r* = 0.124, *P* = 0.014, respectively) (Table 2). Serum concentrations of L and Z were positively correlated with MPOV (*r* = 0.422, *P* < 0.001 and *r* = 0.285, *P* < 0.001, respectively) (Table 2). Regression plots are shown in Figure 2. There were no other significant correlations between MPOV and the clinical and demographic variables shown in Table 1, including medical diagnosis, L/Z dietary intake, smoking status, and sex.

**Table 2:** Macular Pigment Volume Correlations

Variable	Correlation	*P* Value
Age	0.178	<0.001
Education	0.124	0.014
BMI	−0.156	0.002
Serum L	0.422	<0.001
Serum Z	0.285	<0.001
MPOD 0.23	0.695	<0.001
MPOD 0.51	0.769	<0.001
MPOD 0.98	0.855	<0.001
MPOD 1.76	0.906	<0.001

Serum L, serum concentrations of lutein; Serum Z, serum concentrations of zeaxanthin.

MPOV was positively correlated to MPOD at the four measured eccentricities, with the strongest agreement at 1.76° (*r* = 0.906, *P* < 0.001) (Table 2).

### Linear Regression Analysis

Using multiple linear regression analysis, serum concentrations of L and Z, education level, and age were found to be positive predictors of MPOV, whereas BMI was found to be a negative predictor of MPOV (*P* < 0.001).

## Discussion

Given the now-accepted importance of measuring MP for both research and clinical settings, it is essential that the measurement and reporting of MP are standardized for comparison across research laboratories and clinical practices. Here, we propose MPOV as a suitable value for reporting MP, where MPOV is the numeric integration of MPOD values within a radius of a given retinal eccentricity.

The current study supports part-validation of this approach by comparing MPOV with known biological and lifestyle predictors of MP. Specifically, MPOV was positively correlated with serum concentrations of its constituent carotenoids, as expected and comparable with previous reports.^33^,^39^,^40^ This finding is important as it confirms the relationship between the exact molecules in serum and target tissue concentrations of the carotenoids in the macula. Given that carotenoid serum concentrations are obtained from a validated assay technique (HPLC, with known units [μmol/L]), confirming an agreement between these variables helps to validate this method. Of note, some previous studies reporting on MP have demonstrated weak correlations between MP and serum carotenoid concentrations.^33^ However, the results from this study show stronger correlations between serum carotenoid concentrations and MPOV than previously reported using subjective methods (nonimaging-based systems) and for imaging techniques reporting only at a single eccentricity.^41^ The current finding is consistent with other studies,^42^ as confirmed by Conrady et al.,^33^ also using the Spectralis MPOD module, where serum carotenoid concentrations showed a stronger correlation when a broader area of MP was assessed and reported. As expected, in our study MPOV exhibited a strong correlation with MPOD at all eccentricities; however, the strength of the agreement increased with increasing eccentricity.

In the current study, serum L and Z concentrations, BMI, education level, and age were found to be significant predictors of MPOV. Of note, our findings are in agreement with previous studies reporting on determinants of MP.^43^–^45^ For example, it has been shown that BMI is inversely related to MP, where possible explanations can be summarized as follows: body fat may act as a reservoir and compete for uptake by fat-soluble target tissues, given the fat soluble nature of these micronutrients. Second, it is known that obesity results in increased oxidative stress and consequently lower MP. Finally, education has been clearly shown to be a predictor of MP as it is likely related to nutrition and lifestyle habits.^46^

In our sample, there were no significant differences between groups of subjects with different medical diagnoses (e.g., subjects with AD versus control subjects). We believe this lack of significance may be due to the sample size of the individual categories, given that previous work by us^47^ has shown that AD present lower levels of MP compared with control subjects.

MPOV is a measure that enables the assessment of a composite of the three macular carotenoids distributed throughout different anatomic subregions of the macula (i.e., foveola, parafovea, perifovea).^48^ This is one of the reasons why reporting MP in terms of volume provides a full representation of the MP profile. In other words, MPOV relates MP density together with its distribution within the macula, providing a measurement of the total MP across all eccentricities. The MPOV measurement includes values not only at each eccentricity but also within each eccentricity, avoiding the need to estimate volume by interpolating between eccentricities, as is the case with other techniques (e.g., HFP).^49^,^50^

Considering that MP is not symmetric across the macular area, measuring its quantity at one single point leads to misrepresentation of the totality of MP across the macula. For example, two patients may have identical MP values at a central eccentricity, but different MP profiles (one patient with a narrow profile and one patient with an atypical profile; Fig. 1). If only central MP is reported, it is not possible to appreciate the difference in MP between these two subjects. Several such examples presented in this study, where two patients with identical MP values at 0.23° of eccentricity (see subject A and subject B, both with an MPOD of 0.52° at 0.23°) have drastically different MPOV values (4728 and 8404, respectively) (Fig. 1). In addition, other techniques not only measure MP at a single eccentricity but they fail to note that they use a single meridian. These other techniques use point probes and may not accurately measure MPOD, because it is averaged over all directions. The MPOV approach in the current study integrates over all meridians, which is an improvement over a single-point probe. This integration over all meridians averages over all meridians — in itself is an advantage over other techniques, making it superior in that respect. Also, as an imaging-based method, the Spectralis MPOD module output provides valuable information on the reliability of the measurement in particular subjects. For example, in some patients with advanced dry AMD, patchy geographic atrophy generates an irregular background autoflourescence causing standard deviations and ranges enlarge to an extent that MPOD and MPOV measurements lose accuracy.

This discrepancy of MP between studies mentioned above, is further exaggerated after MP supplementation. Studies have shown that carotenoid supplementation changes are noticed in the parafovea (probably due to the high concentration of L in the perifovea).^33^,^51^ This may explain why some studies have failed to show any significant increases in MP following carotenoid supplementation. These studies report MP at only central eccentricities and use a close-in reference point (e.g., at 5° or 6° of eccentricity), where it is assumed that beyond that eccentricity MP is optically undetectable,^52^ when in fact MP may be present in higher concentrations at these eccentricities (especially following carotenoid supplementation).^33^ In this study, the reference point was set at 7° for consistency and to allow for comparison with previous studies using subjective techniques to measure MP (Fig. 1). It should be noted, however, that some centers have chosen to use 9° as their reference eccentricity when using the Spectralis MPOD module.^33^,^51^ These centers are clinic-based and often do measurements on patients with more severe macular pathology, such as Macular Telangiectasia type 2 (MacTel), Stargardt disease, and advanced AMD than typically encountered at the Waterford site. This is particularly important in MacTel where the macular pigment may redistribute into an anomalous ring extending 5° to 6° from the fovea that may not return to baseline until well past 7°. Thus, even though Spectralis MPOV and MPOD values using 7° or 9° eccentricities are likely to correlate strongly, it is important to explicitly report the chosen reference eccentricity to facilitate comparisons between studies.

Furthermore, the ideal measuring technique should be accurate, precise (in terms of reliability and reproducibility), and allow for such results to be obtained in the appropriate study populations.^53^ The Spectralis MPOD module has been shown to provide accurate and precise MP measurements in various populations.^33^,^54^,^55^ In addition, studies have demonstrated that it is easy to implement in clinical practice,^9^,^34^ where it is more likely to be applied in an ageing population, with vision loss, retinal diseases, diminished attention, and with compromised ocular media.^37^ MP acquisition, as noted by Dennison et al.,^56^ requires little subject compliance and takes just a few minutes to perform. Also, it has been shown that MP measurements with the Spectralis MPOD module are highly reproducible in patients with retinal pathologies, such as AMD, macular edema, and tractional maculopathy of different etiologies (i.e., diabetic maculopathy, epiretinal membrane, and macular hole).^57^ Nonetheless, in these and other conditions, such as extensive geographic atrophy, dense cataracts, recent use of fluorescein intravenously or topically, advanced Stargardt disease, and others, the method fails to be accurate and hence is not appropriate to use; however, most competing techniques fail as well in these situations, so caution must be taken for all techniques. In fact, previous work has looked at the effect of cataract on MP measurement. It has been shown that high-grade cataracts cause underestimation of MP^58^ at all eccentricities, including both MPOD and MPOV, using the Spectralis MPOD module. However, for research purposes, this underestimation can be corrected with different proposed correction factors in order to compare across subjects and/or studies.^58^,^59^ Nonetheless, in clinical practice, comparisons should be done within subjects over time where each individual becomes their own control. In addition, it can be recommended that cataract be graded routinely during measurement of MP in older adults to correct for the impact of cataract on MP measurement.^59^ It is our view that there is no accepted gold standard method for measuring MP.^57^ And although there is no perfect measurement technique, reporting MPOV using the Spectralis MPOD module has fewer limitations than other techniques.

The Spectralis is an expensive device relative to others that provide MP measurements, although it offers the option to also acquire spectral-domain OCT images and other imaging modalities on the same platform. Therefore, the Spectralis MPOD module offers additional information about the details and structure of the macula in relation to MP providing a fuller assessment of MP. Although the device is widely used in ophthalmic clinics as a diagnostic aid and for management of various eye diseases where patients are routinely dilated, the need for pupil dilation prior to data acquisition on the Spectralis MPOD module constitutes another limitation. In addition, the MPOD module's flexibility of acquisition settings, mainly regarding the setting of the reference eccentricity, contributes to variability in data that are not standardized across research institutions and studies. Other issues, like anatomic foveal alignment via OCT, the blue and green laser wavelengths, validity in the setting of compromised RPE AF (i.e. RPE degeneration produces a decreased AF signal), among others, need to be addressed. Furthermore, because the Spectralis MPOD module is still investigational, the ability to analyse the resultant data are not intuitive, standardized, and seamless. An expert panel (authors of this manuscript) are collaborating with Heidelberg Engineering to mitigate these technical limitations and introduce newer ways of displaying macular pigment distributions using three-dimensional rendering or pseudocolor intensity plots.

Finally, several published studies have demonstrated that the Spectralis MPOD module is capable of detecting change in MPOV following carotenoid intervention over time,^33^,^47^,^60^,^61^ which is fundamentally important for both the clinic and research setting, when trying to identify and monitor changes in MP. Future studies should also evaluate functional outcomes in relation to MPOV changes and the sensitivity of MPOV to detect change over time. Indeed, some work has already been done to address this; for example, the Central Retinal Enrichment Supplementation Trial (CREST), where significant increases in MPOV were reported in all supplemented subjects, which correlated with improvement in visual function outcomes.^60^

The Spectralis MPOD module offers the ability to objectively acquire MPOV measurements with good repeatability, reproducibility, and accuracy. This combined with the MPOV measurement that takes into account the MP profile within a given area in the macula rather than at discrete eccentricities, offers a robust and reliable technique to assess the MP levels in research and clinical settings. Of note, interpretation and comparison between subjects or studies adds complexity when considering other MP variables, such as distribution patterns and macular disorders, which need to be considered regardless of the technique used.

In summary, MPOV provides a total description of MP and its distribution across the macula. The measurement with Spectralis MPOD module is easy to perform, compared with subjective techniques, the results are reliable and reproducible, and comparable to known predictors of MP. In order to fully derive meaning from scientific studies and to successfully implement this MP measurement technique in research and clinical settings, it is essential to standardise a method to assess and report MP. Such standardization is essential to health recommendations with respect to MP and its role on visual function and retinal disease. Therefore, MPOV measured using the Spectralis MPOD module should be considered as the preferred method for evaluating MP, especially for future clinical trials and research investigations.

## References

[1] Bone RA, Landrum JT, Friedes LM (1997). Distribution of lutein and zeaxanthin stereoisomers in the human retina. *Exp Eye Res*.

[2] Bone RA, Landrum JT, Hime GW, Cains A, Zamor J (1993). Stereochemistry of the human macular carotenoids. *Invest Ophthalmol Vis Sci*.

[3] Wishart DS, Feunang YD, Marcu A HMDB 4.0: the human metabolome database for 2018. *Nucleic Acids Res.*.

[4] Nolan JM, Meagher K, Kashani S, Beatty S (2013). What is meso-zeaxanthin, and where does it come from?. *Eye (Lond)*.

[5] Bernstein PS, Li B, Vachali PP (2016). Lutein, zeaxanthin, and meso-zeaxanthin: the basic and clinical science underlying carotenoid-based nutritional interventions against ocular disease. *Prog Retin Eye Res*.

[6] Remington LA, Remington LA (2012). Chapter 4 - Retina. *Clinical Anatomy and Physiology of the Visual System (Third Edition)*.

[7] Hogan MJ, Alvarado JA, Weddell JE (1971). *Histology of the Human Eye; an Atlas and Textbook*.

[8] Hirsch J, Curcio CA (1989). The spatial resolution capacity of human foveal retina. *Vision Res*.

[9] Lima VC, Rosen RB, Farah M (2016). Macular pigment in retinal health and disease. *Int J Retina Vitreous*.

[10] Bird AC, Bok D (2018). Why the macula?. *Eye (Lond)*.

[11] Williams RA, Brody BL, Thomas RG, Kaplan RM, Brown SI (1998). The psychosocial impact of macular degeneration. *Arch Ophthalmol*.

[12] Shah P, Schwartz SG, Gartner S, Scott IU, Flynn HW (2018). Low vision services: a practical guide for the clinician. *Ther Adv Ophthalmol*.

[13] Scott IU, Smiddy WE, Schiffman J, Feuer WJ, Pappas CJ (1999). Quality of life of low-vision patients and the impact of low-vision services. *Am J Ophthalmol*.

[14] Li B, Ahmed F, Bernstein PS (2010). Studies on the singlet oxygen scavenging mechanism of human macular pigment. *Arch Biochem Biophys*.

[15] Ramkumar HL, Tuo J, Shen DF (2013). Nutrient supplementation with n3 polyunsaturated fatty acids, lutein, and zeaxanthin decrease A2E accumulation and VEGF expression in the retinas of Ccl2/Cx3cr1-deficient mice on Crb1(rd8) background. *J Nutr*.

[16] Snodderly DM, Brown PK, Delori FC, Auran JD (1984). The macular pigment. 1. Absorbance spectra, localization, and discrimination from other yellow pigments in primate retinas. *Invest Ophthalmol Vis Sci*.

[17] Chew EY, Clemons TE, Sangiovanni JP (2014). Secondary analyses of the effects of lutein/zeaxanthin on age-related macular degeneration progression: AREDS2 report No. 3. *JAMA Ophthalmol*.

[18] Akuffo KO, Beatty S, Peto T (2017). The impact of supplemental antioxidants on visual function in nonadvanced age-related macular degeneration: a head-to-head randomized clinical trial. *Invest Ophthalmol Vis Sci*.

[19] Tanprasertsuk J, Mohn ES, Matthan NR (2019). Serum carotenoids, tocopherols, total n-3 polyunsaturated fatty acids and n-6/n-3 polyunsaturated fatty acid ratio reflect brain concentrations in a cohort of centenarians. *J Gerontol A Biol Sci Med Sci*.

[20] Vishwanathan R, Schalch W, Johnson EJ (2016). Macular pigment carotenoids in the retina and occipital cortex are related in humans. *Nutr Neurosci*.

[21] Vishwanathan R, Iannaccone A, Scott TM (2014). Macular pigment optical density is related to cognitive function in older people. *Age Ageing*.

[22] Power R, Coen RF, Beatty S (2018). Supplemental retinal carotenoids enhance memory in healthy individuals with low levels of macular pigment in a randomized, double-blind, placebo-controlled clinical trial. *J Alzheimers Dis*.

[23] Nolan J, Mulcahy R, Power R, Moran R, Howard A (2018). Nutritional intervention to prevent Alzheimer's disease: potential benefits of xanthophyll carotenoids and omega-3 fatty acids combined. *J Alzheimers Dis*.

[24] Hammond BR, Wooten BR, Smollon B (2005). Assessment of the validity of in vivo methods of measuring human macular pigment optical density. *Optom Vis Sci*.

[25] Howells O, Eperjesi F, Bartlett H (2011). Measuring macular pigment optical density in vivo: a review of techniques. *Graefes Arch Clin Exp Ophthalmol*.

[26] Davis MD, Gangnon RE, Lee LY (2005). The Age-Related Eye Disease Study severity scale for age-related macular degeneration: AREDS Report No. 17. *Arch Ophthalmol*.

[27] Randolph C, Tierney MC, Mohr E, Chase TN (1998). The Repeatable Battery for the Assessment of Neuropsychological Status (RBANS): preliminary clinical validity. *J Clin Exp Neuropsychol*.

[28] Guo QH, Cao XY, Zhou Y, Zhao QH, Ding D, Hong Z (2010). Application study of quick cognitive screening test in identifying mild cognitive impairment. *Neurosci Bull*.

[29] Bucks RS, Ashworth DL, Wilcock GK, Siegfried K (1996). Assessment of activities of daily living in dementia: development of the Bristol Activities of Daily Living Scale. *Age Ageing*.

[30] Bucks RS, Haworth J (2002). Bristol Activities of Daily Living Scale: a critical evaluation. *Expert Rev Neurother*.

[31] Tuijl JP, Scholte EM, de Craen AJ, van der Mast RC (2012). Screening for cognitive impairment in older general hospital patients: comparison of the Six-Item Cognitive Impairment Test with the Mini-Mental State Examination. *Int J Geriatr Psychiatry*.

[32] Perry A, Rasmussen H, Johnson EJ (2009). Xanthophyll (lutein, zeaxanthin) content in fruits, vegetables and corn and egg products. *J Food Compost Anal*.

[33] Conrady CD, Bell JP, Besch BM (2017). Correlations between macular, skin, and serum carotenoids. *Invest Ophthalmol Vis Sci*.

[34] Howells O, Eperjesi F, Bartlett H (2011). Measuring macular pigment optical density in vivo: a review of techniques. *Graefes Arch Clin Exp Ophthalmol*.

[35] Nolan JM, Stringham JM, Beatty S, Snodderly DM (2008). Spatial profile of macular pigment and its relationship to foveal architecture. *Invest Ophthalmol Vis Sci*.

[36] Hammond BR, Wooten BR, Snodderly DM (1997). Individual variations in the spatial profile of human macular pigment. *J Opt So Am A Opt Image Sci Vis*.

[37] Delori FC (2004). Autofluorescence method to measure macular pigment optical densities fluorometry and autofluorescence imaging. *Arch Biochem Biophys*.

[38] Meagher KA, Thurnham DI, Beatty S (2013). Serum response to supplemental macular carotenoids in subjects with and without age-related macular degeneration. *Br J Nutr*.

[39] Nolan JM, Stack J (2007). O OD, Loane E, Beatty S. Risk factors for age-related maculopathy are associated with a relative lack of macular pigment. *Exp Eye Res*.

[40] Nolan JM, Stack J, O'Connell E, Beatty S (2007). The relationships between macular pigment optical density and its constituent carotenoids in diet and serum. *Invest Ophthalmol Vis Sci*.

[41] Nolan JM, Stack J, O'Connell E, Beatty S (2007). The relationships between macular pigment optical density and its constituent carotenoids in diet and serum. *Invest Ophthalmol Vis Sci*.

[42] Fujimura S, Ueda K, Nomura Y, Yanagi Y (2016). Preliminary analysis of the relationship between serum lutein and zeaxanthin levels and macular pigment optical density. *Clin Ophthalmol*.

[43] Bovier ER, Lewis RD, Hammond BR (2013). The relationship between lutein and zeaxanthin status and body fat. *Nutrients*.

[44] Power R, Prado-Cabrero A, Mulcahy R, Howard A, Nolan JM (2019). The role of nutrition for the aging population: implications for cognition and Alzheimer's disease. *Annu Rev Food Sci Technol*.

[45] Nolan J, O'Donovan O, Kavanagh H (2004). Macular pigment and percentage of body fat. *Invest Ophthalmol Vis Sci*.

[46] Moran R, Nolan JM, Stack J (2016). Non-dietary correlates and determinants of plasma lutein and zeaxanthin concentrations in the Irish population. *J Nutr Health Aging*.

[47] Nolan JM, Loskutova E, Howard AN (2014). Macular pigment, visual function, and macular disease among subjects with Alzheimer's disease: an exploratory study. *J Alzheimers Dis*.

[48] Trieschmann M, van Kuijk FJ, Alexander R (2008). Macular pigment in the human retina: histological evaluation of localization and distribution. *Eye (Lond)*.

[49] Kirby ML, Beatty S, Loane E (2010). A central dip in the macular pigment spatial profile is associated with age and smoking. *Invest Ophthalmol Vis Sci*.

[50] Kirby ML, Galea M, Loane E, Stack J, Beatty S, Nolan JM (2009). Foveal anatomic associations with the secondary peak and the slope of the macular pigment spatial profile. *Invest Ophthalmol Vis Sci*.

[51] Obana A, Tanito M, Gohto Y, Okazaki S, Gellermann W, Bernstein PS (2015). Changes in macular pigment optical density and serum lutein concentration in Japanese subjects taking two different lutein supplements. *PLoS One*.

[52] Korobelnik JF, Rougier MB, Delyfer MN (2017). Effect of dietary supplementation with lutein, zeaxanthin, and ω-3 on macular pigment: a randomized clinical trial. *JAMA Ophthalmol*.

[53] Rohrig B, du Prel JB, Blettner M (2009). Study design in medical research: part 2 of a series on the evaluation of scientific publications. *Dtsch Arztebl Int*.

[54] Obana A, Gellermann W, Gohto Y (2018). Reliability of a two-wavelength autofluorescence technique by Heidelberg Spectralis to measure macular pigment optical density in Asian subjects. *Exp Eye Res*.

[55] Howells O, Eperjesi F, Bartlett H (2011). Measuring macular pigment optical density in vivo: a review of techniques. *Graefes Arch Clin Exp Ophthalmol*.

[56] Dennison JL, Stack J, Beatty S, Nolan JM (2013). Concordance of macular pigment measurements obtained using customized heterochromatic flicker photometry, dual-wavelength autofluorescence, and single-wavelength reflectance. *Exp Eye Res*.

[57] You QS, Bartsch DU, Espina M (2016). Reproducibility of macular pigment optical density measurement by two-wavelength autofluorescence in a clinical setting. *Retina*.

[58] Obana A, Gohto Y, Sasano H (2018). Grade of cataract and its influence on measurement of macular pigment optical density using autofluorescence imaging. *Invest Ophthalmol Vis Sci*.

[59] Akuffo KO, Nolan JM, Stack J (2016). The impact of cataract, and its surgical removal, on measures of macular pigment using the Heidelberg Spectralis HRA+OCT MultiColor Device effect of cataract on Spectralis MP measurement. *Invest Ophthalmol Vis Sci*.

[60] Nolan JM, Power R, Stringham J (2016). Enrichment of macular pigment enhances contrast sensitivity in subjects free of retinal disease: Central Retinal Enrichment Supplementation Trials - Report 1. *Invest Ophthalmol Vis Sci*.

[61] Nolan JM, Loskutova E, Howard A (2015). The impact of supplemental macular carotenoids in Alzheimer's disease: a randomized clinical trial. *J Alzheimers Dis*.

